# Target 2035 – update on the quest for a probe for every protein

**DOI:** 10.1039/d1md00228g

**Published:** 2021-12-03

**Authors:** Susanne Müller, Suzanne Ackloo, Arij Al Chawaf, Bissan Al-Lazikani, Albert Antolin, Jonathan B. Baell, Hartmut Beck, Shaunna Beedie, Ulrich A. K. Betz, Gustavo Arruda Bezerra, Paul E. Brennan, David Brown, Peter J. Brown, Alex N. Bullock, Adrian J. Carter, Apirat Chaikuad, Mathilde Chaineau, Alessio Ciulli, Ian Collins, Jan Dreher, David Drewry, Kristina Edfeldt, Aled M. Edwards, Ursula Egner, Stephen V. Frye, Stephen M. Fuchs, Matthew D. Hall, Ingo V. Hartung, Alexander Hillisch, Stephen H. Hitchcock, Evert Homan, Natarajan Kannan, James R. Kiefer, Stefan Knapp, Milka Kostic, Stefan Kubicek, Andrew R. Leach, Sven Lindemann, Brian D. Marsden, Hisanori Matsui, Jordan L. Meier, Daniel Merk, Maurice Michel, Maxwell R. Morgan, Anke Mueller-Fahrnow, Dafydd R. Owen, Benjamin G. Perry, Saul H. Rosenberg, Kumar Singh Saikatendu, Matthieu Schapira, Cora Scholten, Sujata Sharma, Anton Simeonov, Michael Sundström, Giulio Superti-Furga, Matthew H. Todd, Claudia Tredup, Masoud Vedadi, Frank von Delft, Timothy M. Willson, Georg E. Winter, Paul Workman, Cheryl H. Arrowsmith

**Affiliations:** Institute of Pharmaceutical Chemistry, Goethe University Frankfurt Frankfurt 60438 Germany; Structural Genomics Consortium, BMLS, Goethe University Frankfurt Frankfurt 60438 Germany susanne.mueller-knapp@bmls.de; Structural Genomics Consortium, University of Toronto Toronto Ontario M5G 1L7 Canada Cheryl.Arrowsmith@uhnresearch.ca; University of Toronto Toronto Ontario M5G 1L7 Canada; Department of Data Science, The Institute of Cancer Research London SM2 5NG UK; CRUK ICR/Imperial Convergence Science Centre London SM2 5NG UK; Cancer Research UK Cancer Therapeutics Unit, The Institute of Cancer Research London SM2 5NG UK; Monash Institute of Pharmaceutical Sciences, Monash University Parkville Victoria 3052 Australia; School of Pharmaceutical Sciences, Nanjing Tech University No. 30 South Puzhu Road Nanjing 211816 People's Republic of China; Research and Development, Bayer AG, Pharmaceuticals 42103 Wuppertal Germany; Centre for Medicines Discovery, University of Oxford Old Road Campus Research Building, Roosevelt Drive Oxford OX3 7DQ UK; Merck KGaA Darmstadt Germany; Alzheimer's Research UK Oxford Drug Discovery Institute, Centre for Medicines Discovery, University of Oxford Oxford OX3 7FZ UK; Institut Recherches de Servier 125 Chemin de Ronde 78290 Croissy France; Discovery Research, Boehringer Ingelheim 55216 Ingelheim am Rhein Germany; Early Drug Discovery Unit (EDDU), Montreal Neurological Institute-Hospital, McGill University Montreal QC Canada; School of Life Sciences, Division of Biological Chemistry and Drug Discovery, University of Dundee James Black Centre Dundee UK; Structural Genomics Consortium, UNC Eshelman School of Pharmacy Chapel Hill NC USA; Lineberger Comprehensive Cancer Center, Department of Medicine, School of Medicine, University of North Carolina at Chapel Hill Chapel Hill NC 27599 USA; Structural Genomics Consortium, Department of Medicine, Karolinska University Hospital and Karolinska Institutet Stockholm Sweden; Nuvisan Innovation Campus Berlin GmbH Müllerstraße 178 13353 Berlin Germany; Center for Integrative Chemical Biology and Drug Discovery, Division of Chemical Biology and Medicinal Chemistry, Eshelman School of Pharmacy, University of North Carolina at Chapel Hill Chapel Hill NC 27599 USA; Institute for Protein Innovation Boston MA USA; National Center for Advancing Translational Sciences, National Institutes of Health Rockville Maryland 20850 USA; Medicinal Chemistry, Global R&D, Merck Healthcare KGaA Frankfurter Straße 250 64293 Darmstadt Germany; Takeda California 9625 Towne Centre Drive San Diego California 92121 USA; Science for Life Laboratory, Department of Oncology-Pathology Karolinska Institutet Stockholm Sweden; Institute of Bioinformatics and Department of Biochemistry and Molecular Biology, University of Georgia Athens GA USA; Genentech, Inc. 1 DNA Way South San Francisco California 94080 USA; Department of Cancer Biology and Chemical Biology Program, Dana-Farber Cancer Institute 450 Brookline Ave Boston MA 02215 USA; CeMM Research Center for Molecular Medicine of the Austrian Academy of Sciences Vienna Austria; European Molecular Biology Laboratory, European Bioinformatics Institute Wellcome Genome Campus, Hinxton Cambridgeshire CB10 1SD UK; Strategic Innovation, Global R&D, Merck Healthcare KGaA Frankfurter Straße 250 64293 Darmstadt Germany; Kennedy Institute of Rheumatology, NDORMS, University of Oxford UK; Neuroscience Drug Discovery Unit, Research, Takeda Pharmaceutical Company Limited Fujisawa Kanagawa Japan; Chemical Biology Laboratory, Center for Cancer Research, National Cancer Institute, National Institutes of Health Frederick MD USA; LMU Munich, Department of Pharmacy, Chair of Pharmaceutical and Medicinal Chemistry 81377 Munich Germany; Discovery Network Group, Pfizer Medicine Design Cambridge MA 02139 USA; Drugs for Neglected Diseases initiative 15 Chemin Camille Vidart Geneva 1202 Switzerland; AbbVie North Chicago Illinois USA; Global Research Externalization, Takeda California, Inc. 9625 Towne Center Drive San Diego CA 92121 USA; Department of Pharmacology & Toxicology, University of Toronto Toronto Ontario M5S 1A8 Canada; Research and Development, Bayer AG, Pharmaceuticals 13353 Berlin Germany; Structural & Protein Sciences, Discovery Sciences, Janssen Research & Development 1400 McKean Rd Spring House PA 19477 USA; Division of Rheumatology, Department of Medicine Solna, Karolinska University Hospital and Karolinska Institutet Stockholm Sweden; Center for Physiology and Pharmacology, Medical University of Vienna Vienna Austria; School of Pharmacy, University College London London WC1N 1AX UK; Diamond Light Source Ltd Harwell Science and Innovation Campus Didcot OX11 0QX UK; Department of Biochemistry, University of Johannesburg Auckland Park 2006 South Africa; Research Complex at Harwell Harwell Science and Innovation Campus Didcot OX11 0FA UK; Princess Margaret Cancer Centre Toronto Ontario M5G 1L7 Canada

## Abstract

Twenty years after the publication of the first draft of the human genome, our knowledge of the human proteome is still fragmented. The challenge of translating the wealth of new knowledge from genomics into new medicines is that proteins, and not genes, are the primary executers of biological function. Therefore, much of how biology works in health and disease must be understood through the lens of protein function. Accordingly, a subset of human proteins has been at the heart of research interests of scientists over the centuries, and we have accumulated varying degrees of knowledge about approximately 65% of the human proteome. Nevertheless, a large proportion of proteins in the human proteome (∼35%) remains uncharacterized, and less than 5% of the human proteome has been successfully targeted for drug discovery. This highlights the profound disconnect between our abilities to obtain genetic information and subsequent development of effective medicines. Target 2035 is an international federation of biomedical scientists from the public and private sectors, which aims to address this gap by developing and applying new technologies to create by year 2035 chemogenomic libraries, chemical probes, and/or biological probes for the entire human proteome.

## Abstract 2

Twenty years after the publication of the first draft of the human genome, our knowledge of the human proteome is still fragmented. Target 2035 aims to develop a pharmacological modulator for every protein in the human proteome to fill this gap.

## Graphical abstract

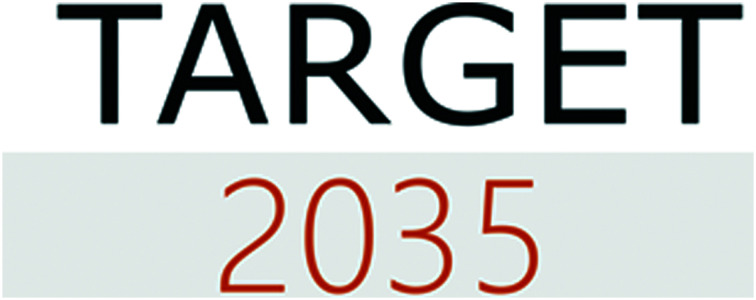

## Target 2035: conceptual framework

The Target 2035 concept arose from discussions among scientists at the Structural Genomics Consortium (SGC) and like-minded colleagues in industry, government and academia^2,5–7^ who recognised the slow progress towards understanding and exploiting the plethora of human proteins (‘dark proteome’) despite their suspected or potential role in disease states.^1–4^ This large untapped reservoir may provide new targets to address unmet medical needs. We recognised the lack of tools to study protein function as the main obstacle to progress in this area. Therefore, we launched Target 2035 as an ambitious open science initiative to discover and make available chemogenomic libraries,^8–11^ chemical probes, and/or functional antibodies for nearly all human proteins by the year 2035.^2,12^

**Fig. 1:**
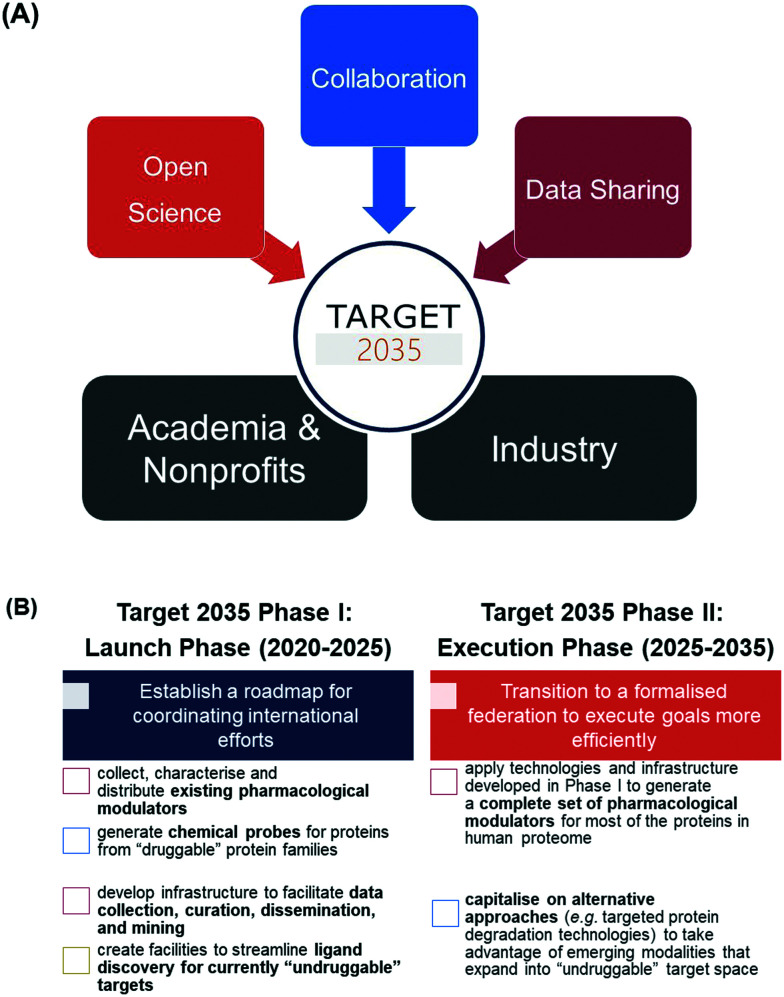
Conceptual framework and strategic priorities for Target 2035. (A) Target 2035 integrates principles of open science, collaboration, and data sharing between scientists from academic, non-profit and industrial institutions. (B) Target 2035 will be executed in two phases (phase I and II), with clearly defined strategic priorities aligned with the ultimate goal to develop chemogenomic libraries, pharmacological and biochemical tool compounds (chemical probes), and/or functional antibodies for nearly all human proteins by the year 2035.

Target 2035 provides a strategy for the global research community to work together to create the technologies and research tools required to interrogate the function and therapeutic potential of all human proteins. We are especially interested in the discovery of pharmacological modulators (chemical probes and chemogenomic compounds), given that availability of such compounds is known to have a transformative effect on the ability to study protein biology and target proteins for drug discovery purposes.^2^ It is expected that the resulting research tools and knowledge will significantly enhance our ability to translate the tremendous recent advances in genomics into new medicines. Importantly, the Target 2035 model is founded on open science, collaboration, and data sharing between interested scientists from both the public and private sectors (Fig. 1A).

The SGC has assumed an initial leadership and organisational role and, in consultation with the broader community, formulated key short-term and long-term strategic priorities (Fig. 1B). The short-term priorities (phase I) focus on establishing a roadmap around which international groups of like-minded scientists can assemble and collaborate towards the following goals: (1) collecting, characterising, and distributing existing pharmacological modulators; (2) generating novel chemical probes for ‘druggable’ proteins; (3) developing a centralised infrastructure to facilitate data collection, curation, dissemination, and mining; and (4) creating centralised facilities to streamline ligand discovery for currently ‘undruggable’ targets. Long-term priorities would build on the achievements of phase I to transition into a more formalised federation and accelerate efforts towards creating solutions for the ‘dark proteome’ (Fig. 1B).

Here, we provide an update on the progress towards establishing phase I of Target 2035 as a robust collaborative network of scientists to develop and apply new technologies towards generating specific pharmacological modulators for all human proteins.

### Outreach activities towards engaging the global research community

Target 2035 is currently an informal alliance of biomedical scientists, and its success will require much wider participation, collaboration, and innovation from the global research community.^2^ Thus, a key challenge facing Target 2035 in this launch phase is increasing awareness of the initiative in the biomedical research community, encouraging participation, and enabling knowledge exchange and collaborative opportunities. The website https://www.target2035.net is the main gateway currently being used to share information and communicate with the research community. Additionally, we have published an initial announcement of the initiative,^2^ launched a series of webinars (see below) and leverage social media (#Target2035) as strategies to inform, engage and nucleate a Target 2035 community.

### Webinar series

In order to engage the research community, Target 2035 hosts free (open) monthly webinars amenable to multiple global time zones. The webinars are also recorded and available online at https://www.target2035.net/archived-recordings and on https://www.youtube.com/channel/UCpl3xd4P7aYedOg6uw53hpg; they are valuable resources for the scientific community including trainees in medicinal chemistry and chemical biology. The kick-off webinars in November 2020 featured key chemical biology and drug discovery opinion leaders who discussed the challenges and impact of Target 2035. Subsequent monthly webinars have focused on specific themes including targeted protein degradation and proximity-induced pharmacology, covalent ligand screening, computational and artificial intelligence (AI) methods for ligand discovery and chemoproteomic methods.^13^

Overall, feedback from the scientific community has been positive, with new collaborations arising from research highlighted in the webinars. Further, the Target 2035 community is active on social media and online tools (#Target2035, https://en.wikipedia.org/wiki/Target_2035), with much discussion of webinars, speakers, and recent publications. By facilitating the online conversation, we hope to gain an increase in awareness and involvement and continue to build the community.

## New initiatives and research projects

Various relevant large-scale initiatives are already underway, several with the expressed objective of contributing to Target 2035. These consortia and groups are generally multidisciplinary, international and open access. Notably many groups – such as members of the Illuminating the Druggable Genome (IDG)^4^ project whose output is published in Pharos,^14^ the SGC and EUbOPEN (described below) – are taking a protein family approach, which is an efficient and practical way to tackle the human proteome.^2,15^ These efforts are often building on other enabling initiatives, where key reagents and protocols for an entire target class are being generated. The ReSOLUTE initiative, for example, is already well on its way to unlock solute carriers (SLCs) for chemical probe discovery by the community.^16^ The outputs of ReSOLUTE include robust assays for most SLCs in the genome, as well as the creation of other empowering tools, such as thousands of tailored cell lines, and making all data, protocols, and reagents publicly available through their portal or open distributors (re-solute.eu). The IDG project, supported by the NIH Common Fund,^17^ is developing chemical tools, assays, expression data, interaction maps, and knock-out mice for many dark members^18^ of the highly druggable GPCR, kinase, and ion channel protein families. All data, reagents, and tools generated by IDG are accessible through its portal.^19^ The IDG is also developing a suite of informatics tools that can be used to interrogate the biological function of these dark proteins and their potential role in human diseases.

Due to space restrictions, we will highlight here just a few of the additional initiatives that directly impact Target 2035 goals.

### EUbOPEN

**Fig. 2:**
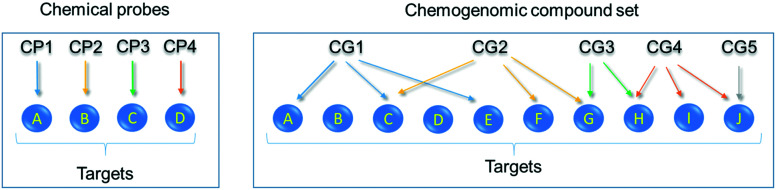
Relationship between chemical compounds and their targets for chemical probes *versus* chemogenomic compound sets. Chemical probes (CP)s are small molecules that are potent, selective and cell-active. Ideally, a chemical probe negative control is identified and used in parallel in phenotypic assays. A chemical probe ideally binds to a single target but can also be useful if it targets just a small number (∼2–3) of very closely sequence-related proteins. A chemogenomics library (CGL) comprises chemogenomic (CG) compounds of different chemotypes for each target; the chemotypes have complementary selectivity profiles, and preferably distinct modes of action (such as orthosteric or allosteric modulation). In the schematic, a CGL was screened against targets A–J yielding phenotypic data for 8 targets. These data are interpreted in the context of the magnitude of the phenotypic response, the mode of action (colour of arrow) and the CG chemical structures and accompanying characterisation.

Having identified the need for high quality chemical probes for the research community, the EUbOPEN partnership of excellence (www.EUbOPEN.org) came together to ‘**E**nable and **U**nlock **b**iology in the **OPEN**’ as part of the Innovative Medicines Initiative (IMI).^20^ EUbOPEN aims to generate the largest freely available set of high-quality chemical modulators for human proteins. These compounds will complement existing chemical probe resources such as the SGC's kinase, epigenetic and donated chemical probes collections.^21–23^ The EUbOPEN compound collections will include chemical probes for high impact target areas relating to human health, including solute carriers (SLCs) and ubiquitin ligases (E3s), where high-quality small-molecule binders have historically been lacking compared to other families *e.g.* protein kinases. It will also compile a chemogenomic library (CGL) of ∼4–5000 compounds covering one third of the current druggable genome (Fig. 2). All compounds will be comprehensively characterised by defining their selectivity, potency, and cellular activity. The compounds will be annotated with activity data from a series of established and novel biochemical and cell-based assays, including many derived from primary patient cells. The development of new technologies to discover and characterise chemogenomics compounds and chemical probes is a core component of the project.

EUbOPEN partners include scientists from academia and industry developing openly available research reagents and exploring new protein target classes. Of key importance, the EUbOPEN project has a strong commitment to open data and open science, which will accelerate the dissemination of knowledge and project outputs, including chemogenomic library sets, chemical probes, assay protocols and associated research. To boost the project, EUbOPEN partners interact extensively with other large consortia (*e.g.* ReSOLUTE,^16^ Illuminating the Druggable Genome,^24^ Open Targets^25^) thus minimising the duplication of effort and maximizing global coordination. Finally, sustainability of the project's resources will be ensured through partnerships with chemical vendors and cheminformatics/database providers, which will ensure the long-term availability of the new chemical tool sets, all associated profiling data and established protocols. By working openly, and engaging the wider research community, this project will deliver pharmacological tool compounds for previously understudied targets, contributing to the Target 2035 campaign, and form the foundation for future efforts to generate chemical modulators for the entire druggable genome.

The SGC laboratory at the University of Toronto has launched a pilot project, *Target 2035: **WDR proteins as a technology test-bed for illuminating the dark proteome***. WD40 repeat (WDR) domains are protein–protein interaction domains comprising one of the largest and most disease-associated protein families in the human proteome.^26,27^ This open science, industry-partnered programme is comparing different experimental strategies for progressing WDR targets from gene to chemical modulator, including AI-guided methods to accelerate discovery of new chemical tools for this under-explored protein family.

### Open chemistry networks projects

The creation of such a large number of chemical modulators will require a significant amount of synthetic medicinal and organic chemistry to design, make and test compounds that are not commercially available. The SGC's new Open Chemistry Networks initiative aims to create opportunities for the community to drive the development of these novel probes through a highly distributed, open chemistry network in which chemical resources (compounds, ideas, laboratory space and time) are contributed on a patent-free, open access basis in return for biological evaluation of those compounds by the Target 2035 network, as well as exposure to the educational opportunities afforded by Target 2035. All crowdsourced chemistry inputs and Target 2035-generated assay data will be openly available to the research community without restrictions, under a novel set of open terms, analogous to a Creative Commons Attribution 4.0 International license for chemistry, including for use by participants towards co-authored publications and grant proposals. Based on prior experience with such distributed chemistry,^28^ different cohorts of scientists are likely to be interested in different types of projects. The synthetic routes will range from simpler (for example, the synthesis of close analogues of a hit compound by straightforward coupling reactions) to more demanding modifications (for example, significant changes to a compound's core scaffold to optimise specific properties). The former might be of interest to undergraduate student cohorts while the latter may be a more time-intensive project suitable for a MSc or PhD thesis. Each project will be led by a science team that could involve a variety of stakeholders/contributors such as: 1) the discoverer of the hit; 2) a pharmaceutical industry mentor, tasked with ensuring the project is kept on track and key data are collected; and 3) a champion acting as the project manager in areas such as managing meetings, the project ‘To Do list’ and community outreach. The online infrastructure needed (*e.g.* online lab notebook,^29^ chemical registration system, project coordination website) will be easy to use and freely accessible to all participants. To ensure a clear description of project status and to ensure a connection between project activity and paper authorship, a novel form of ‘living paper’ will operate for each project: a series of preprints, where each ‘version’ is posted upon reaching a project milestone. Progress towards implementing this system has already been made and the first project has been posted.^30^ Others will come online as Target 2035 accelerates.

### EU-OPENSCREEN^31^

EU-OPENSCREEN^31^ is an open screening platform that offers access to a broad range of technologies and tools for the systematic screening of chemical inhibitors. Scientists from academia and industry can implement their screening projects at EU-OPENSCREEN's partner laboratories using the European Chemical Biology Library (ECBL), a diversity compound collection of 140 000 commercially available as well as academic compounds, crowdsourced through chemical biology networks. All generated data and tool compounds are made available in an open access database for use by the wider life science as well as drug discovery communities.

### FAIRplus data sharing

As outlined above, Target 2035 aims can only be reached with mass collaboration, including openness and data sharing. Given the vast number of different types of biological data sets produced during the chemical probe development process, and differences in data management between institutions, data outputs can be highly variable. The FAIRplus project aims to change and sustain the data management culture by providing tools to ensure that data produced follows community standards and ontologies, along with well-defined data dissemination pathways. As an example, EUbOPEN's mandate is to share its project outputs in a sustainable manner. Therefore, the project is committed to ensuring that all outputs are compliant with FAIR principles:^32^ data is findable, accessible, interoperable, and reusable. To support this effort, EUbOPEN works directly with the FAIRplus IMI consortium who are providing advice and support to ensure the outputs can be used by the research community. The collaboration is part of EUbOPEN's drive to develop sustainable infrastructures and feeds the FAIRplus vision of making all life science data FAIR.

### The Chemical Probes Portal

The Chemical Probes Portal (Portal; https://www.chemicalprobes.org) is a long-standing resource for the biomedical research community that provides expert-curated information on the quality of chemical probes, and enables users to identify the best probes for a biological experiment or study.^5^ The Portal was very recently re-launched to provide the community with an enhanced and extended user-friendly interface and integrate chemical compound information from multiple relevant databases to enable user decision-making in terms of the right chemical modulator for a given target and a given biological context. Although there are several complementary online resources available that automatically score the quality of probes using computational approaches,^33–38^ such as Probe Miner,^39^ thus far the Portal is the only expert-curated resource.^33^ The Portal currently features more than 600 compounds covering 300 targets and draws on more than 170 chemical biology experts to provide three reviews for each probe, commenting on their characterisation data and properties and providing recommendations on their usefulness, flaws, and strengths. While the majority of probes so far are inhibitors or antagonists, the Portal provides guidelines and information pages about new types of chemical modulators such as PROTACs and molecular glue degraders, and recommendations and guidelines about evaluating their performance.^40^ Importantly, the Portal also contains a list of historical but still prevalent compounds that typically are non-selective or not sufficiently potent compared to other available chemical probes in order to discourage their use when interrogating the biological activity of specific targets. The new user interface will facilitate the submission of probes and reviews, streamlining the process to facilitate an increase in target coverage.

### Considerations for the open sharing of chemical matter

The discovery of new chemical matter is often associated with IP protection, based on the expectation that IP protection is required for further translation, application, or exploitation of the discovery. However, IP protection can also be a barrier to collaboration and open sharing of information or materials, and therefore requires new approaches, creativity and novel ways of thinking. A number of innovative approaches have been chosen by different stakeholders. Broadly, they fall into two categories. The first, is for a single ‘closed’ discovery team (such as a single lab or company) to establish their IP position for a chemical probe (to be patented or not patented), and then share the chemical probe with the community for unrestricted research use. For example, as part of the DCP program, pharmaceutical companies provide the SGC with a license to make data and compounds available to the public, which is also extended to selected trusted commercial vendors in order to guarantee a sustainable supply of the chemical probe matter as well as quality and structural identity of the distributed compounds. In a similar strategy, Boehringer Ingelheim makes some of their well-characterized compounds available *for free via* their opnMe program,^41^ where researchers can order molecules directly *via* the website for unencumbered research use. The second, alternative approach is that taken by the SGC for the discovery of novel chemical probes. In this scenario multi-institutional groups collaborating on a given chemical probe project agree, *in advance*, that the output of their research will be an unencumbered public domain tool. This enables a more open and rapid exchange of information and materials, and does not preclude the future development of therapeutic products based on the initial chemical probe. Such products may emerge through public and philanthropic investment to advance the initial probe into later-stage development^42,43^ or through a more traditional proprietary drug development paradigm, for example by advancing patented, optimized derivatives of the initial probe structure^44^ or novel chemical matter developed through further studies enabled by the probe.

## Future outlook

Since the launch of Target 2035 in 2019, we have witnessed the success of the open science model in the global fight against the COVID-19 pandemic. The swift sharing of SARS-CoV2 viral sequences^45^ and other data has led to extraordinarily rapid development of new vaccines^46^ and small molecule antiviral trials.^47–50^ A recent example is the fast development of anti-viral small molecules against the SARS-CoV-2 protease, as part of an initiative called the COVID Moonshot.^51^ The rapid release of the SARS-CoV-2 Mpro protease crystal structure^52^ and subsequent crystallography-based fragment screening campaign at the Diamond Light Source XChem facility set the starting point for this drug discovery endeavour.^53^ In collaboration with laboratories based in several continents, some of these compounds have been characterised and progressed into anti-viral probes that are shortly entering clinical trials. Similarly, global open collaborative initiatives in drug discovery addressing different SARVS-CoV-2 targets have also significantly advanced the development new anti-virals.^50,54^ The events of the past year and a half have demonstrated how scientists in academia and industry can work together around the world at a pace and scale to address important biomedical problems with open science driving the early stages of drug/vaccine research.

Target 2035 aspires to replicate this success but on a human-proteome scale, which will require building a large robust scientific community that works together using open science principles to achieve its ambitious goals and to avoid redundancies and wasting of scarce resources. We have already made significant progress in developing the underlying infrastructure, *e.g.*, with respect to data handling and mining.

Future plans include expansion of the Target 2035 webpage to become a gateway for information sharing and communication among interested scientists. This will include a collaboration marketplace where researchers will be able to share information on their research projects and identify potential collaborators with complementary expertise, infrastructure, and capabilities. We invite those interested to engage with the current Target 2035 team and consider getting involved. Novel technologies, such as machine learning and high throughput chemistry, proteome-wide analyses, and collaborative research approaches will need to be developed to accelerate the rate of ligand discovery and characterisation. The challenges of finding a high quality tool for any protein of interest by 2035 are immense. However, if we work together and share the results freely, we will succeed in reaching this goal as seen from the success of the Human Genome Project, also a highly collaborative, global effort. We are optimistic that major breakthroughs are around the corner and that we will enable high quality biological data linking specific protein targets with phenotypic outcomes and new therapeutic hypotheses for tomorrow's medicines.

## Conflicts of interest

A. A. A., B. A.-L., I. C., and P. W. are employees of the Institute of Cancer Research (ICR) which operates a Rewards to Inventors scheme whereby employees of the ICR may receive financial benefit following commercial licensing of their research. P. W. is a consultant/scientific advisory board member for Nextech Invest Ltd, Storm Therapeutics, Astex Pharmaceuticals, Black Diamond Therapeutics, CV6 and Vividion Therapeutics, and holds stock in Chroma Therapeutics, NextInvest, and Storm Therapeutics. P. W. is also a non-executive director of Storm Therapeutics and the Royal Marsden NHS Trust; a board member and executive director of the non-profit Chemical Probes Portal; and a former employee of AstraZeneca. P. W. has received research funding from Vernalis, Astex Therapeutics, Merck KGaA, BACIT/Sixth Element Capital/CRT Pioneer Fund. B. A.-L. is/was a consultant/scientific advisory board member for GSK, Open Targets, Astex Pharmaceuticals, Nuvectis Pharma and Astellas Pharma, and is an ex-employee of Inpharmatica Ltd. A. A. A., B. A.-L., and P. W. have been instrumental in the creation/development of canSAR and Probe Miner. B. A.-L. was instrumental in the creation of ChEMBL and is a director of the non-profit Chemical Probes Portal. I. C. is/was a consultant to Epidarex LLP, AdoRx Therapeutics, and Enterprise Therapeutics, and is a director of the non-profit Chemical Probes Portal. I. C. has received research funding from Astex, Merck KGaA, Janssen Biopharma, Monte Rosa Therapeutics, and Sixth Element Capital/CRT Pioneer Fund. I. C. holds stock in Monte Rosa Therapeutics AG and is a former employee of Merck Sharp & Dohme. M. K. is a paid consultant for Life Science Editors (LSE). A. C. receives or has received sponsored research support from Almirall, Amphista therapeutics, Boehringer Ingelheim, Eisai, Nurix therapeutics, and Ono Pharmaceuticals. A. C. is a scientific founder, shareholder, and consultant of Amphista therapeutics. A. R. L. has consulted for Astex Therapeutics and has received research funding from Novo Nordisk. S. Ku. is a co-founder and shareholder of Proxygen GmbH and Solgate GmbH. G. E. W. is a founder and shareholder of Proxygen and Solgate Therapeutics. He is on the Research Review Committee of Almirall and coordinates a research collaboration between CeMM and Pfizer. S. F. reports equity ownership and membership on the Meryx Board of Directors and consulting or SAB relationships with Artios, Astex, Cullgen, Design Therapeutics, Flare, Mitokinin, Pathios, ReViral and eFFector. This communication reflects the views of the authors and neither IMI nor the European Union, EFPIA or any Associated Partners are liable for any use that may be made of the information contained herein.

## Supplementary Material
